# Exhaustion and performativity: the relationship between psychosocial factors and burnout syndrome in companies

**DOI:** 10.47626/1679-4435-2025-1427

**Published:** 2025-08-25

**Authors:** Euda Kaliani Gomes Teixeira Rocha, Hélio Soares de França, Carolina Pontual Cardoso de Vasconcelos, Valdézio José dos Santos Neto, Júlia Maria Leandro Teles

**Affiliations:** 1 Department of Physchology, Universidade Federal de Pernambuco, Recife, PE, Brazil

**Keywords:** occupational health, occupational diseases, mental health, burnout, psychological, saúde ocupacional, doenças profissionais, saúde mental, esgotamento psicológico

## Abstract

Current forms of management are based on the meritocratic discourse of self-entrepreneurship, seting people against each other and, at the same time, against themselves, in an exhausting logic of performativity and an exacerbated “cult” of performance and productivity. This logic is now revered as a new ethos, that of competitiveness between workers, which inevitably leads to illness through the daily overtaxing of human limits. Herein, we discuss burnout syndrome from a sociogenic perspective, i.e., as a severe consequence of contemporary work processes on worker health. The World Health Organization’s recognition of burnout syndrome as a work-related mental illness opened debate with global repercussions. As information about the syndrome has disseminated, certain aspects have been trivialized, with analysis tending to focus on personal weakness, which individualizes the treatment and prevention of symptoms. This type of analysis, although important for highlighting the debate, overlooks the socio-historical and psychosocial aspects of burnout. Thus, the discussion was based on an analysis of the sociogenic aspects of the syndrome with a view to identifying and preventing the syndrome, caring for affected workers, and improving work environments.

## Abstract (pt)

As formas de gestão contemporâneas se estabelecem a partir do discurso meritocrático do empreendedorismo de si mesmo, colocando as pessoas umas contra as outras e, ao mesmo tempo, contra si mesmas, numa lógica extenuante de performatividade e de culto exacerbado ao desempenho e à produtividade. Esta lógica passa a ser reverenciada como um novo *ethos*, o da competitividade entre trabalhadores/as, gerando inevitáveis adoecimentos pelo extrapolamento cotidiano dos limites do ser humano. O presente artigo objetiva trazer a discussão sobre a síndrome de *burnout* a partir de uma perspectiva sociogênica, no intuito de tematizá-la como consequência severa dos processos contemporâneos de trabalho para a saúde dos trabalhadores. O reconhecimento da síndrome de *burnout* como uma doença mental relacionada ao trabalho pela Organização Mundial da Saúde trouxe uma abertura ao debate, rapidamente repercutindo em escala global. Com a difusão de informações sobre a síndrome, alguns aspectos tendem a ser banalizados e as análises tendem a focar em fragilidades pessoais, recaindo em considerações que individualizam as formas de lidar com o adoecimento e de tratar os sintomas. Esse tipo de análise, embora importante para visibilizar o debate, dificulta uma compreensão ampla que envolva os aspectos sócio-históricos e psicossociais que envolvem a síndrome de *burnout.* Portanto, conduzimos a discussão a partir da análise dos aspectos sociogênicos da síndrome, com o intuito de colaborar com a forma de ver e compreendê-la, no sentido da identificação, da prevenção e do cuidado das pessoas e dos ambientes de trabalho afetados.

## INTRODUCTION

The film They Shoot Horses, Don’t They?,^1^ set in the context of the economic depression of the 1930s, when the majority of Americans faced unemployment and increasingly precarious living conditions, illustrates exhaustion as a spectacle through a macabre competition. In search of a 1,500 dollar prize, the contestants participate in a dance marathon. Despite complete physical and mental exhaustion, they continue competing and, the more they perform, the more tips they earn, without realizing the danger and exploitation they have been subjected to. The emcee, who would now be considered a “motivational coach”, stirs up the audience’s expectations about the next couple to give up or collapse while simultaneously encouraging the dancers through catchphrases such as “prosperity is just around the corner”. Quack doctors are employed, not for the participants’ well-being, but rather to maintain minimum functionality so that they can return as quickly as possible to the same degrading and inhumane system.

Although fictional, this iconic film can be considered symbolic of the spirit of our times, especially when workers, in order to remain viable, are required to multitask in a juggling act that can lead to extreme fatigue, exhaustion, and illness. If, on the one hand, this juggling act aims to meet the countless demands, requirements and performance expectations, on the other, it is still necessary to deal with the pressure on mental and physical health, while making time for leisure and maintaining social relationships, etc.^2^ In this context, we will discuss burnout syndrome a consequence of exhausting work processes, given that they have been encouraged in contemporary times.

Recognized in 2022 by the World Health Organization as an occupational disease, burnout syndrome can be defined as a psychological disorder marked by a high state of emotional tension, stress, and exhaustion resulting from work situations that exceed individual capabilities, leading to severe exhaustion.^3^

Discourse on the subject does not always assume a critical view that considers the syndrome from the perspective of workers, organizations, and society more broadly. Because the motivational speech of meritocracy is based on a hyper-individualistic perspective, treating burnout syndrome as a banal and natural side effect of progress while shifting responsibility for it to workers, we propose another approach: understanding it as a social symptom whose costs fall not only on the affected individuals, but on society as a whole, including organizations.

Throughout this article, we will engage in a critical discussion of burnout as a disorder driven by the cult of performance and productivity at any cost.^4^ We believe that a society in which the law of profit is placed above everything and everyone is inhumane, with no room for compassion or the sense of collectivity that makes us human.^5^

### THE ETHICAL-CIVILIZING CONDITION OF OUR TIMES AND PRODUCTIVITY AS A MORAL VALUE

Work plays a fundamental role in our lives, and it is through work that we develop our skills and potential. Subjectively mediated by work, we are both the creators and creatures of ourselves.^6,7^ However, in the capitalist and neoliberal organization of society, work assumes a solely mercantile character, becoming the main means of survival and subsistence.^8,9^ As a relationship based solely on the sale of labor, work’s meaning is lost, emptying it.^9,10,11^

Hence, the current world of work pits people against each other and, at the same time, against themselves by seeking performance, perfection, and efficiency in function of unlimited productivity. Such goals are worshipped as moral values, while simultaneously generating a mechanism to intensify competition among workers.

In a political-social configuration where individualism and meritocratic discourse are the foundations of work, rather than collectivity and citizenship, success is seen as an isolated effort, which radically disregards interdependence, a sense of belonging, and social responsibility.^5^ Due to its intensity, productivist logic is not constrained by human limitations.^6^

In such a scenario, the idea that happiness and well-being are achieved through sacrifice, self-denial, and individual effort is well received. Thus, individuals perform work that tests their own limitations in search of chimerical and superhuman levels of performance, as if they were invulnerable and highly adapted to change and potentially degrading and sickening work contexts.^6^

From its early stages to the present day, the goal of capitalism has been extreme efficiency, achieving maximum production with minimum resources, whether human or otherwise. Frederick Taylor (1855-1915), the creator of the first model of scientific labor management, known as Taylorism, was one of the first to rationalize labor activities using techniques that aimed to extract maximum efficiency and productivity through organizations. Although his model dates back to the second phase of the Industrial Revolution (1870-1914), it continues to influence the way work is perceived and organized to this day. His goal was to increase productivity while reducing work hours through the extreme division of labor activities, the standardization of movement, and the time allotted for performing tasks, which are intense simplifications that dispense with worker qualifications.^11^

Following the logic of maximum exploitation and fragmentation of work processes, Fordism, as the method developed by Henry Ford (1863-1947) became known, introduced two mechanisms that widened the gap between the skill of workers and control over them: the conveyor belt and interchangeable parts. With the mass production line, tasks were segmented and managed to such an extent that they became extremely simple, so that time began to be meticulously controlled to achieve maximum productivity.^11^

In late capitalism, productive restructuring, which began in the 1970s, led to a series of changes that liquefied labor and social rights.^12^ One example of which is the dismantling of public and social policies, such as health and education, which then cease to be the full responsibility of the State and begin being sold as commodities by the private sector.

### THE IDEOLOGIES OF PRODUCTION METHODS AND DEHUMANIZATION

In the 1960s and 1970s, Taylorism-Fordism began to decline and strategies for addressing difficulties and maintaining capital hegemony were developed mainly by alignment with the Japanese production method known as Toyotism, which was developed by Taiichi Ohno (1912-1990). From then on, streamlining and flexibility took precedence, which led to a series of losses for the working class, since, for this new system to be viable, legal and institutional support from the State was necessary.^9,11^

Thus, flexibility became the keystone of all work relationships and processes, giving rise to the era of flexible accumulation based on the following methods: outsourcing, just-in-time, kaizen, kanban, total quality control circle, bonus remuneration, partial contracts, etc.^9,11^ The logic of Toyotism not only regulates production processes but has spread to the service sector as well, causing massive human suffering through unrealistic goals.

If, in the process of producing goods, Toyotism works toward accurate, lean, synchronized, and lossfree production, in the service sector (especially sales), control is unfeasible in the face of demand, which is external and spontaneous. The distorted logic used to set targets, in alignment with flexible relationships (remuneration, working hours, rights, coverage, employment relationship, etc.), results in harmful interactions for workers, based on management through stress and harassment.

In this process, labor unions lose strength, and workers are left to their own devices in the midst of rising unemployment, having to submit to intensified work and constant adaptation to an increasingly insecure and globalized economy.^8,9,12^ Media and social networks have become a means of transmiting the values and atributes desired for the new economic and social configuration: eagerness for activity, individualism, maximum performance, and productivity.

Thus, a new ethic, another ethos is established, a way of life based on efficiency and effectiveness.^13^ Paradoxically, despite the security it offers, this ethos provides no support whatsoever. On the contrary, it encourages an increasingly hyperactive and accelerated subjectivity, constantly affected by the discomfort arising from the pressure of never doing enough and not living up to performance demands, despite one’s best effort.^5,13,14^

Contemporary malaise is caused by an excess of positivity, a characteristic of today’s tired society,^15^ in which discipline becomes liquid.^12^ Thus, the enemy becomes a kind of ghost that cannot be perceived directly because it is embodied in the subject itself. The pressure to perform and overwork then becomes a vicious circle of self-exploitation that, despite creating a sense of freedom and autonomy, leads to exhaustion. Due to its coercive nature, this same freedom becomes violent, as it reflexively refers to the subject who performs, consequently resulting in psychological disorders, such as burnout syndrome. Considered this way, illness does not necessarily emerge from excessive responsibility and proactivity, but rather from performance as the new order of society.^15^

Digital platform work has become increasingly visible since the COVID-19 pandemic. This type of non-binding labor relationship has again transformed the morphology of work, increasing and intensifying the number of workers in precarious digital employment.^15^ In this new paradigm, control of work rhythm, time, and remuneration is determined by algorithm in the logic of “the zero contract”. The zero contract dismantles obligations and rights, reducing remuneration strictly to the time used to perform a task. In other words, in “uberized” work, the Taylorist-Fordist dream reaches its peak, since the porosity of work time is eliminated with each process. For example, the time in which workers wait to be called call is not computed in remuneration, only the minutes during which the work activity is performed.

Thus, work loses its material form, circumscribed by an intermittent time and a speed dictated by the digital application.^13^ In this format, there is no previously agreed upon or regulated definition of working hours, nor agreement about the distribution of work or remuneration.^16^

Thus, collapsing and meaningless work is the corollary to intense labor mediated by a society that encourages excessive productivity, pressure for results, performance, and competitiveness, the human cost of which can translate into burnout syndrome and other disorders. However, burnout must not be seen an unavoidable outcome common to all workers. On the contrary, healthy and decent work should be considered universally possible. Neoliberalism must not be understood as an economic system alone, but as an ethos, a way of life defined by a semantics of discomfort, accompanied by appropriate techniques for managing the discomfort.^16^

With this new ethos based in human subjectivities, neoliberal control models benefit, gaining speed and intensity, as society embraces unconstrained individualism regarding work.^16^

There is an urgent need for analysis of this issue in a broad and contextualized way, so that therapeutic actions – whether pharmacological, psychiatric or psychotherapeutic – are not seen as sufficient or as a solution for individual workers. On the contrary, these strategies should be integrated into an approach that focuses on work processes arising from a social, economic, and political environment that catalyzes subjectivity, exhaustion, and illness.

### CHARACTERIZATION OF BURNOUT SYNDROME

Burnout syndrome is a specific condition of work-related mental illness and should not be used to describe situations in other areas of life. The syndrome was included in both the tenth and eleventh editions of the International Classification of Diseases, with the definition being more detailed in the latter:

Burnout is a syndrome conceptualized as resulting from chronic workplace stress that has not been successfully managed. It is characterised by three dimensions: 1) feelings of energy depletion or exhaustion; 2) increased mental distance from one’s job, or feelings of negativism or cynicism related to one’s job; and 3) a sense of ineffectiveness and lack of accomplishment.^3^

Burnout syndrome involves a wide variety of symptoms that are related to the worsening of stressful situations. Such symptoms can be categorized as affective, cognitive, physical, attitudinal, behavioral, or social:^17,18,19^

1. Affective: emotional exhaustion, depressive mood and sudden mood changes, feelings of helplessness, low self-esteem, irritation, hostility, lack of professional fulfillment, high frustration, and emotional detachment.
2. Cognitive: difficulty concentrating, memory loss, low creativity, and sensorimotor symptoms (tics, agitation).
3. Physical: fatigue, gastrointestinal problems, headaches, insomnia, tiredness, sweating, tremors, shortness of breath, and high blood pressure spikes.
4. Atitudinal: depersonalization, dehumanization, insensitivity, indifference, coldness, and cynicism.
5. Behavioral: the use and abuse of drugs (alcohol, pharmaceuticals, or illegal drugs), hyperactivity, accidents, absenteeism and presenteeism, neglect, detachment, and low productivity.
6. Social: problems with clients, colleagues, superiors, and subordinates; isolation.

Burnout syndrome is expressed through the symptoms listed above (with variations), which are indicative of stressful situations that, due to unhealthy human relationships and working conditions, culminate in a complex disorder that affects several aspects of the human psyche. However, these symptoms, whether combined or in isolation, are insufficient to diagnose burnout syndrome. To confirm diagnosis, the three dimensions described in the World Health Organization definition must be expressed: emotional exhaustion, depersonalization, and low emotional fulfillment.^18,19^ We will discuss each of them below to describe their characteristics in the clearest way possible.

Emotional exhaustion from work is defined as a constant feeling of insufficient emotional resources to deal with the challenges of the work routine. We point out that this does not equate to physical exhaustion, but rather exhaustion on an emotional and affective level.

Depersonalization, a perceived reduction in one’s ability to work, commonly involves doubts about one’s professional abilities, uncertainty about previously mastered skills, and fear about one’s ability to perform common tasks. Depersonalization plays an important role in personality symptoms. We can venture to say that this is the central dimension of the syndrome and the main factor responsible for the suffering it entails. It results in a distorted sense of self, which can lead to serious difficulties not only in performing work activities, but in constant doubt and uncertainty about one’s own ability to work (regardless of one’s work history).

The low professional fulfillment dimension refers to high levels of frustration and dissatisfaction at work. There is a tendency to evaluate oneself in a derogatory way. Feelings of unhappiness and dissatisfaction with work are evident and nearly unbearable. This dimension involves sadness and a lack of motivation about work, which can be confused with depression.

Diagnosing burnout syndrome requires three main types of investigation: assessment of the patient’s work and clinical history, clinical examination, and specific psychological instruments. This approach is important to distinguish between work-related stress, depression, and burnout syndrome. Because these symptoms can be similar or even identical, it is essential to investigate the causal link with work.

Treatment involves therapeutic, psychological, and psychiatric dimensions, and may include individual and/or group psychotherapy, yoga or relaxation sessions, or acupuncture, associated or not with antidepressant drugs depending on each case. Regular exercise may also be part of the treatment.

However, caution is required when treating burnout syndrome. First, isolated treatment without changing the work environment will not be as effective since, upon returning from leave, workers will find themselves in the same circumstances that triggered the disorder. Another caveat is that classifying an activity as mentally unhealthy also classifies the human relationships in that particular work environment as unsatisfactory. We also point out that any strategy to improve the work environment is a strategy to prevent not only burnout, but any mental illness triggered by work.

### PSYCHOSOCIAL RISKS AND CONSEQUENCES FOR WORKERS

The World Health Organization’s inclusion of burnout syndrome as an occupational disease led to its correct classification, which also increased the relevance of mental health care for workers and inevitably involved discussion of psychosocial risk at work.

Psychosocial risk includes aspects of work relationships that can cause physical, psychological and/or social harm. In 2021, the International Organization for Standardization developed standard 45003,^20^ which deals with guidelines for managing psychosocial risks. This standard highlights some aspects related to psychosocial risks: the way work is organized, social and human factors at work, characteristics of the work environment and equipment, and dangerous tasks. The standard also states that such risks can cover all types of sectors and organizations, as well as affect all types of tasks and their various employment arrangements.

The psychosocial risks associated with burnout syndrome are diverse. Due to chronic stress arising from the work environment, risks include excessive work demands, a lack of control and autonomy, and inadequate interpersonal relationships in the workplace. Such conditions create a scenario in which workers are exposed to stressors that can culminate in the development of the syndrome.^21^ Lack of social support at work, role ambiguities, and conflicts are also considered significant psychosocial risks. Similarly, low autonomy and lack of support from colleagues and superiors can amplify perceived overload and professional ineffectiveness, central characteristics of the syndrome.^22^ The aforementioned risks can create an environment conducive to burnout, especially among professionals in precarious working conditions and with few opportunities for professional expression or recognition.^22^

Another aspect to be considered in relation to the work environment is the presence of violence, such as moral and sexual harassment, which are recognized as serious psychosocial risks.^23^ Exposure to these risks can not only lead to emotional exhaustion, but also result in severe physical and psychological harm.^23^

Psychosocial risks also affect the physical health of workers.^22^ This is because tense situations at work generate stress, demanding a level of vigilance and effort from the body that is both exhausting and makes homeostatic balance difficult, as reported by Selye.^24^ This wear and tear alters the body’s chemical composition and weakens its structures, making it more vulnerable to disease. Thus, the chronic stress associated with burnout can result in a range of physical conditions, such as sleep disturbances, cardiovascular problems, and a weakened immune system. These physical effects can perpetuate the cycle of burnout, making recovery more difficult and increasing the risk of lost work.

The syndrome can also lead to more serious consequences, considering all the above mentioned factors in addition to worsening mental health and the increasing pressure felt by workers. Social isolation and the loss of meaning in work, often associated with depersonalization, can lead to feelings of hopelessness and despair.

The debate about psychosocial risks highlights aspects that are usually neglected not only in management methods, but also among workers themselves in their daily interactions on the job. Considering these factors and observing potential risk factors is one of the first steps toward identifying, managing, and preventing work-related mental illness.

## CONCLUSIONS

Safe and healthy work environments are not only inalienable rights, but are also necessary for improved productivity and service quality, while insecure and precarious work environments can pose a risk to the mental health of workers. The best way to deal with burnout is to prevent and intervene in factors associated with it. An organization guided by high productivity and competitiveness that uses mechanisms like stress, fear, abusive goals, and harassment risks the health of the people who work there, resulting in a degrading environment.

Throughout this article, we have addressed burnout syndrome not as an individual problem but as a consequence of social labor relations in the neoliberal capitalist context, and it should be considered as such. It is essential for workers, health professionals, employers, and management to avoid trivializing the syndrome.

Some initiatives have great potential for worker well-being and health, such as providing the following elements for the full and healthy exercise of work activities: breaks, safety equipment, ergonomic conditions, professional and personal appreciation, fair wages, recognition, a career progression plan, and respect for the balance between work and personal life. All of these elements contribute to prevention and have a positive impact on mental health.

When analyzing and considering burnout syndrome, it is important to avoid reductionist approaches that place responsibility for the disorder, as well as its treatment, on the worker through coping strategies and individualized interventions. Seeking to explain the disorder based on individual failures or vulnerabilities, and rationalizing that work reveals individual weakness, forces workers into a helpless situation that condemns them to loneliness.

Understanding burnout syndrome and its effects requires analysis focused on work relationships and the work environment. It is a mental disorder with psychological consequences resulting from highly controlled work involving excessive affective, emotional, and intellectual demands.

In addition to individual symptoms, there are sociogenic aspects to burnout syndrome. These aspects must be considered if the phenomenon is to be analyzed comprehensively and judiciously. As mentioned throughout the text, burnout syndrome is a repercussion of currently prevailing labor conditions, in which work demands too much and gives too little in return. This refers not only to remuneration, but to genuine recognition and symbols of affection, respect, and gratitude for the work performed. It also refers to exchanges in which we relate to each other, respecting and recognizing who we are: human beings.
